# Cationic and Anionic Dual Redox Activity of MoS_2_ for Electrochemical Potassium Storage

**DOI:** 10.1021/acsmaterialslett.4c01455

**Published:** 2024-10-09

**Authors:** Ajay Piriya Vijaya Kumar Saroja, Yupei Han, Charlie A. F. Nason, Gopinathan Sankar, Pan He, Yi Lu, Henry R. Tinker, Andrew Stewart, Veronica Celorrio, Min Zhou, Jiayan Luo, Yang Xu

**Affiliations:** † Department of Chemistry, University College London, London WC1H 0AJ, U.K.; ‡ Diamond Light Source, Harwell Science and Innovation Campus, Didcot OX11 0DE, U.K.; § Hefei National Research Center for Physical Sciences at the Microscale, School of Chemistry and Materials Science, University of Science and Technology of China, Hefei, Anhui 230026, China; ∥ State Key Laboratory of Metal Matrix Composites, School of Materials Science and Engineering, Shanghai Jiao Tong University, Shanghai 200240, China

## Abstract

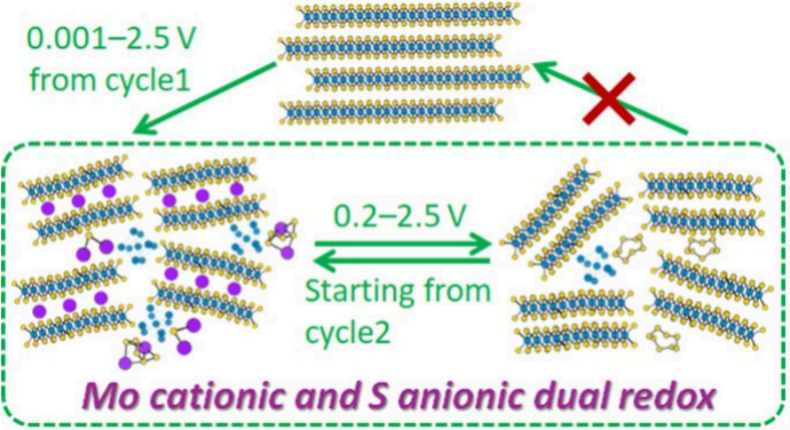

MoS_2_ is regarded as one of the most promising potassium-ion battery (PIB) anodes. Despite the great progress to enhance its electrochemical performance, understanding of the electrochemical mechanism to store K-ions in MoS_2_ remains unclear. This work reports that the K storage process in MoS_2_ follows a complex reaction pathway involving the conversion reactions of Mo and S, showing both cationic redox activity of Mo and anionic redox activity of S. The presence of dual redox activity, characterized in-depth through synchrotron X-ray absorption, X-ray photoelectron, Raman, and UV–vis spectroscopies, reveals that the irreversible Mo oxidation during the depotassiation process directs the reaction pathway toward S oxidation, which leads to the occurrence of K–S electrochemistry in the (de)potassiation process. Moreover, the dual reaction pathway can be adjusted by controlling the discharge depth at different cycling stages of MoS_2_, realizing a long-term stable cycle life of MoS_2_ as a PIB anode.

Two-dimensional transition metal dichalcogenides (2D TMDs) are fundamentally fascinating and chemically versatile materials for a variety of applications.^1,2^ Among various TMDs, molybdenum disulfide (MoS_2_) has been investigated as a promising electrode material for potassium-ion batteries (PIBs) because the wide interlayer spacing (0.62 nm) and weak van der Waals interaction between the S–Mo–S slabs can effectively accommodate large K ions and lower the energy barrier of K-ion diffusion. A great deal of research attention has been directed toward increasing the PIB capacity of MoS_2_ by addressing issues such as poor electronic conductivity and structural instability through carbon coating^3−5^ and phase engineering (1T phase and 1*T*/2H mixed phase).^6,7^ Structural stability can be strengthened by expanding interlayer spacing^8−10^ and tuning the morphology and/or dimensionality of MoS_2_ to relieve the strain in vertical and radial directions during K-ion insertion.^11,12^ However, understanding of the electrochemical mechanism to store K-ions has not been progressed to the same extent as capacity enhancement due to the complexity in phase transition and kinetic processes involved in electrochemical K storage in MoS_2_.^11,13−15^

Electrochemical storage mechanism of alkali-ions in MoS_2_ is strongly affected by the thermodynamic formation energy and polarity of the alkali–metal–sulfur bond. The decomposition enthalpies of AMoS_2_ (A = Li, Na, K) into Mo and A_2_S are −4.46, −3.16, and −3.30 eV f.u.^–1^ for Li, Na, and K, respectively,^16^ indicating the thermodynamic driving force for the conversion reaction to occur decreases from Li to K. Kinetically, bond polarity decreases from the Li–S to K–S bond, which suggests the conversion reaction in the K-MoS_2_ system is more suppressed than the Li-MoS_2_ system. This could result in different mechanisms between K and Li storage in MoS_2_. Even comparing Li and Na storage in MoS_2_, the mechanisms reported in the literature varied from study to study, and diverse conclusions were drawn. It has been reported that MoS_2_ was regenerated after delithiation, accompanied by the structural transition from bulk to nanostructures.^17^ However, the regeneration was not observed in some other studies where partially reversible conversion reaction back to MoS_2_^18^ and even irreversible conversion reaction^19^ were observed. Sodiation in MoS_2_ was reported to be a reversible^16,20^ and an irreversible^21^ conversion process with the structure changing to few layers. Another study^22^ reported MoS_2_ failed to undergo conversion reaction; instead, sodiation caused structural transition forming distorted MoS_*x*_ clusters, with partial regeneration to MoS_2_ after desodiation. There have been very limited studies on K storage in MoS_2_, but vastly different results were seen. Du et al. reported K-ion favored intercalating in MoS_2_ to form KMoS_2_, and the reversible reaction between KMoS_2_ and K_*x*_MoS_2_ was limited only to an intercalation process, which is responsible for K storage.^23^ However, other studies reported a range of discharge products including K_2_S^24^ and a mixture of K_*x*_MoS_2_, K_2_S_5_, and K_2_S,^25,26^ suggesting that K-ion storage in MoS_2_ might undergo a more complex mechanism than the Li and Na counterparts.^22,23,27,28^ Note that the kinetics of K-ion diffusion in MoS_2_ is sluggish due to the large size of the K ion, and this can further increase the complexity of the K-ion storage mechanism.

In this work, we investigated the mechanism of the K storage process in MoS_2_ using commercially available material and a combination of electrochemical, spectroscopic, structural, and morphological characterizations. We revealed the formation of metallic Mo and molecular S at the depotassiated state of MoS_2_, which is due to the irreversible oxidation of the metallic Mo formed at the potassiated state to Mo^4+^ and the resulting oxidation of S^2–^ to S^0^. As a result, a dual reaction pathway consisting of Mo cationic redox activity and S anionic redox activity (i.e., K–S electrochemistry) directed the K storage process in MoS_2_. Moreover, we provided a solution for controlling discharge depth at different cycling stages to realize high cycling stability and rate capability. Unlike the mainstream study of MoS_2_ for PIBs, our investigation reveals interesting simultaneous cationic and anionic redox activity, contributing new insights into design strategies to further enhance the PIB performance of MoS_2_.

Phase purity and chemical composition of commercial MoS_2_ were confirmed by the results of X-ray diffraction (XRD, Figure S1a), Raman (Figure S1b), and X-ray photoelectron spectroscopy (XPS, Figure S1c,d). It shows a sheet-like morphology with an average lateral size of ∼30 μm (Figure S1e,f) and an interlayer spacing of 0.62 nm (inset in Figure S1e), with Mo and S homogeneously distributing across the sheets (Figure S2). We first investigated the galvanostatic charge/discharge (GCD) profiles of the MoS_2_ electrodes cycled in the range of 0.001 to 2.5 V (denoted as Route A in Figure 1a) in the half-cell configuration, as this has been the voltage range (i.e., deep discharge) used in the literature to study MoS_2_ as an anode material of PIBs.^29−32^ Shown in Figure S3a, the first discharge curve exhibited three voltage plateaus, suggesting a multistep potassiation process. XRD patterns (Figure S3b) were obtained for the discharge products at various stages indicated in Figure S3a. At stage II, the (002) peak was broadened and a new peak at 8.9° appeared, which indicates that the initial K-ion intercalation retains the layered structure but expands the interlayer spacing.^33^ Further discharging to stage III caused more K-ion intercalation, as evidenced by the increase in peak intensity at 8.9° and the near disappearance of the original (002) peak. From stage III to IV, a long and stable voltage plateau appeared at ∼0.1 V and both peaks at 14.3° and 8.9° diminished, while a broad peak appeared at 13.4°. This indicates that the layered structure was destroyed and amorphous products formed at the deep discharge condition. At the end of the following charge (stage V), no crystalline product(s) can be detected, suggesting that there was minimal to no restoration of the pristine layered structure. The observations of the GCD profiles via Route A (0.001–2.5 V) directed our attention to the control of discharge depth and its effect on K storage mechanism in MoS_2_. Since the layered structure was retained at >0.2 V potassiation and amorphous discharge products were formed at <0.2 V potassiation, we kept the discharge depth at 0.001 V in the first cycle but changed it to 0.2 V from the second cycle and onward (denoted as Route B: 0.001–2.5 V in the first cycle and 0.2–2.5 V from the second cycle in Figure 1b). Figure 1b shows the GCD profiles via Route B and the XRD patterns of the MoS_2_ electrodes at various stages of the cycles shown in Figure S4. The GCD profiles share similarities with those via Route A, including the three defined plateau regions in the first discharge curve and the sloping curves in the following cycles. The similarities can also be seen from the CV curves via the two routes (Figure S5), showing similar voltages of the reduction and oxidation peaks. No crystalline phase(s) was recovered via Route B and (de)potassiation product(s) remained amorphous judging from the XRD patterns.

**Figure 1:**
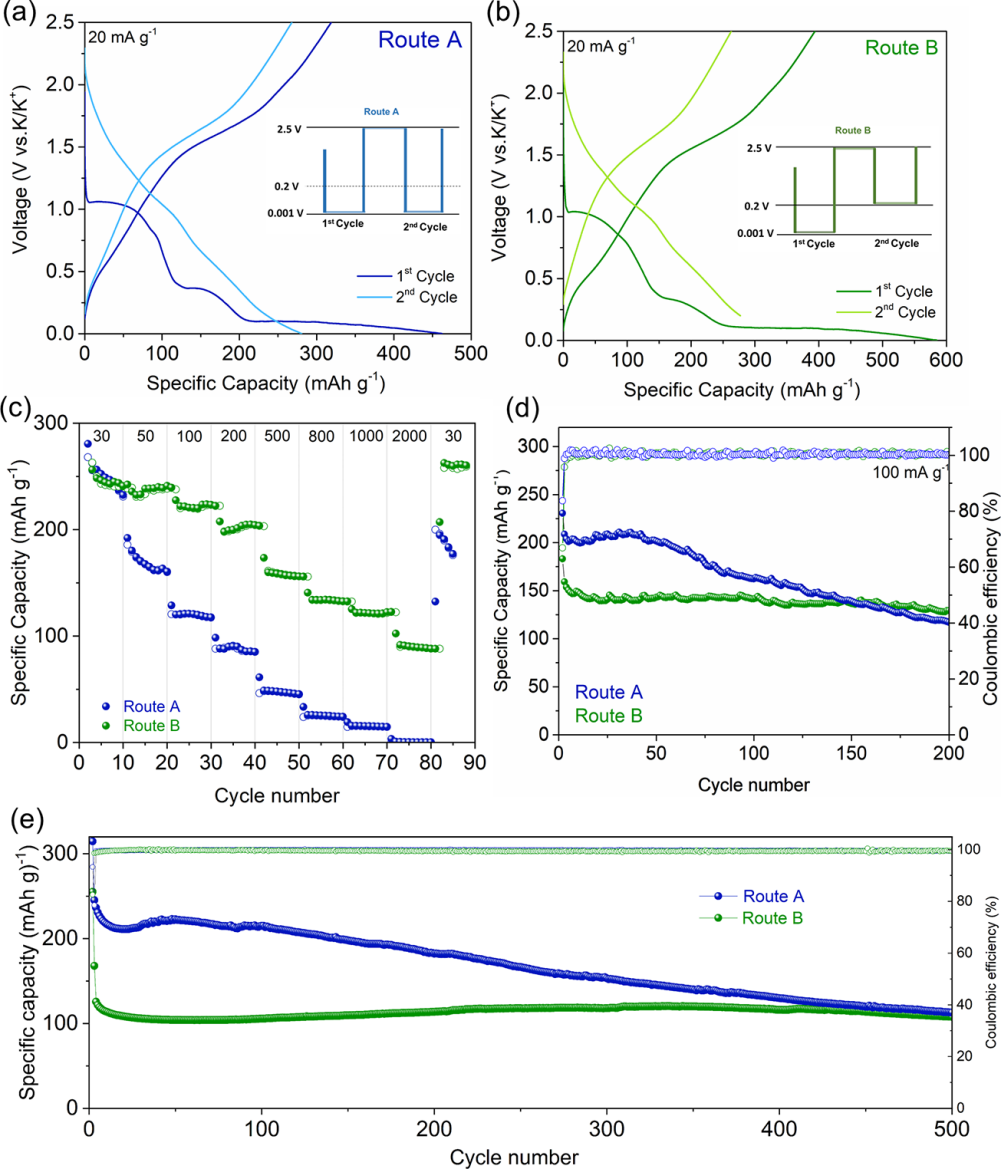
GCD profiles of MoS_2_ via (a) Route A and (b) Route B. Comparison of (c) rate capability and cycle stability at (d) 100 mA g^–1^ and (e) 1 A g^–1^ of MoS_2_ cycled via the two routes.

However, we found that the two routes resulted in a surprisingly different cell performance. First, cycle1 charge and cycle2 discharge capacities via route B were 393 and 273 mAh g^–1^, respectively, at 20 mA g^–1^, being close to the corresponding capacity via route A (318 and 279 mAh g^–1^). It is not difficult to understand cycle1 charge capacity being close, as the two routes underwent the same deep discharge process during the first cycle. However, the similar cycle2 discharge capacity suggests there was extra capacity generated in the 0.2–2.5 V range via Route B to compensate for the capacity loss that would have been gained in the 0.001–0.2 V range via Route A. Second, despite the smaller voltage range from cycle 2 onward, Route B resulted in higher capacities than Route A across all current densities tested for rate capability (Figure 1c). The cell via Route B delivered capacities of 256, 236, 222, 198, 160, 134, 124, and 91 mAh g^–1^ at 30, 50, 100, 200, 500, 800, 1000, and 2000 mA g^–1^, respectively. It retained 263 mAh g^–1^ when returning to 30 mA g^–1^. In contrast, the cell via Route A showed rapid capacity decay and failed at >1 A g^–1^. Third, Route B exhibited much better cycling stability than Route A (Figure 1d). At 100 mA g^–1^, the cell via Route B delivered an initial discharge capacity of 167 mAh g^–1^, 77% of which was retained after 200 cycles. Although the cell via Route A delivered a higher capacity in the initial cycles due to the wider voltage range (0.001–2.5 V), only 54% capacity was retained after 200 cycles, being surpassed by the cell via Route B from cycle154 onward. The same trend was observed at 1 A g^–1^ over 500 cycles (Figure 1e). Route B resulted in 84% capacity retention in comparison to 44% retention via Route A. Fourth, Route B showed a higher Coulombic efficiency (CE) than Route A (Figure 1d). The initial CE of the two routes is ∼65% due to the same discharge depth at 0.001 V. But CE varies in the next few cycles between the two routes, as the discharge depth was different from the second cycle onward. The CE stabilized at ∼99% after 6 cycles for Route A, whereas it reached >99% within 3 cycles for Route B. We are mindful that the PIB performance obtained here is not comparable to some of the best in literature obtained by forming nanocomposites^15,34^ because commercial MoS_2_ in a bulky size was directly used in our work. However, the comparison of the two routes shown here is intriguing because despite the smaller voltage range of Route B than Route A from cycle 2 onward, the former exhibited better rate capability, more stable cycle life, and high capacity over long-term cycling. This indicates that K storage in MoS_2_ may undergo a more complex process than what has been reported in literature, i.e., an intercalation reaction followed by a conversion reaction (possible overlap depending on sample status). Additional reaction(s) may occur simultaneously, and controlling the discharge depth can affect the additional reaction(s), thus affecting the PIB performance of MoS_2_.

We then turned our focus to investigating the change of Mo and S, the two elements in MoS_2_, during the cycles. We combined various spectroscopy techniques including X-ray absorption (XAS), XPS, Raman and UV–vis spectroscopies for the investigation due to the amorphous nature of the discharge and charge products as shown in the previous discussion. The local structural change of the Mo species in MoS_2_ in the first two cycles via Route B was characterized using EXAFS spectra (Figure 2a).^35^Figure 2a shows the Fourier transformed (FT) Mo K-edge EXAFS taken at various states during the first two cycles via Route B. The fitting of the EXAFS spectra is shown in Figure S6. Two strong peaks can be seen at 2.4 and 3.1 Å from pristine MoS_2_, representing the Mo–S interaction in the first coordination shell and the Mo–Mo interaction in second coordination shell of 2H-MoS_2_, respectively (Figure 2a).^36^ When discharged to 0.2 V, the Mo–Mo (3.1 Å) peak of MoS_2_ decreased, and the decrease in intensity was caused by structural transformation of MoS_2_ to K_*x*_MoS_2_.^18^ Further potassiation to 0.001 V caused a significant decline in the amplitude of the Mo–S peak and a strong amplitude of the Mo–Mo interaction at 2.8 Å, the latter corresponding to metallic Mo seen in the Mo foil reference. This proves the occurrence of the conversion reaction that results in the loss of layered structure and the reduction of Mo^4+^ to Mo^0^.^18^ This is also confirmed from the best-fit results of the coordination number (CN) provided in Table S1. The CN of Mo–S bond when discharged to 0.2 V was close to the CN at the pristine state (5.7 vs 5.9). Further discharging to 0.001 V caused a decrease in CN to 2.9, signaling that ∼50% of the phase fraction of MoS_2_ is converted to Mo metal. At the end of the first charge, Mo–S peak was resumed due to the restoration of the MoS_2_ structure upon depotassiation, but not to the same extent of pristine state. Interestingly, the Mo–Mo peak was present alongside the Mo–S peak with a similar amplitude, which clearly suggests the partial oxidation of Mo^0^ back to Mo^4+^ and the coexistence of metallic Mo with the restored MoS_2_. Mo–S CN restored to 5.9, and Mo–Mo metal CN reduced to 1.9 (Table S1). The next cycle in the 0.2–2.5 V range showed reversible peak changes, where Mo–S decreased upon potassiation and increased upon depotassiation, whereas Mo–Mo was present throughout (de)potassiation and became even stronger than Mo–S after second depotassiation.

**Figure 2:**
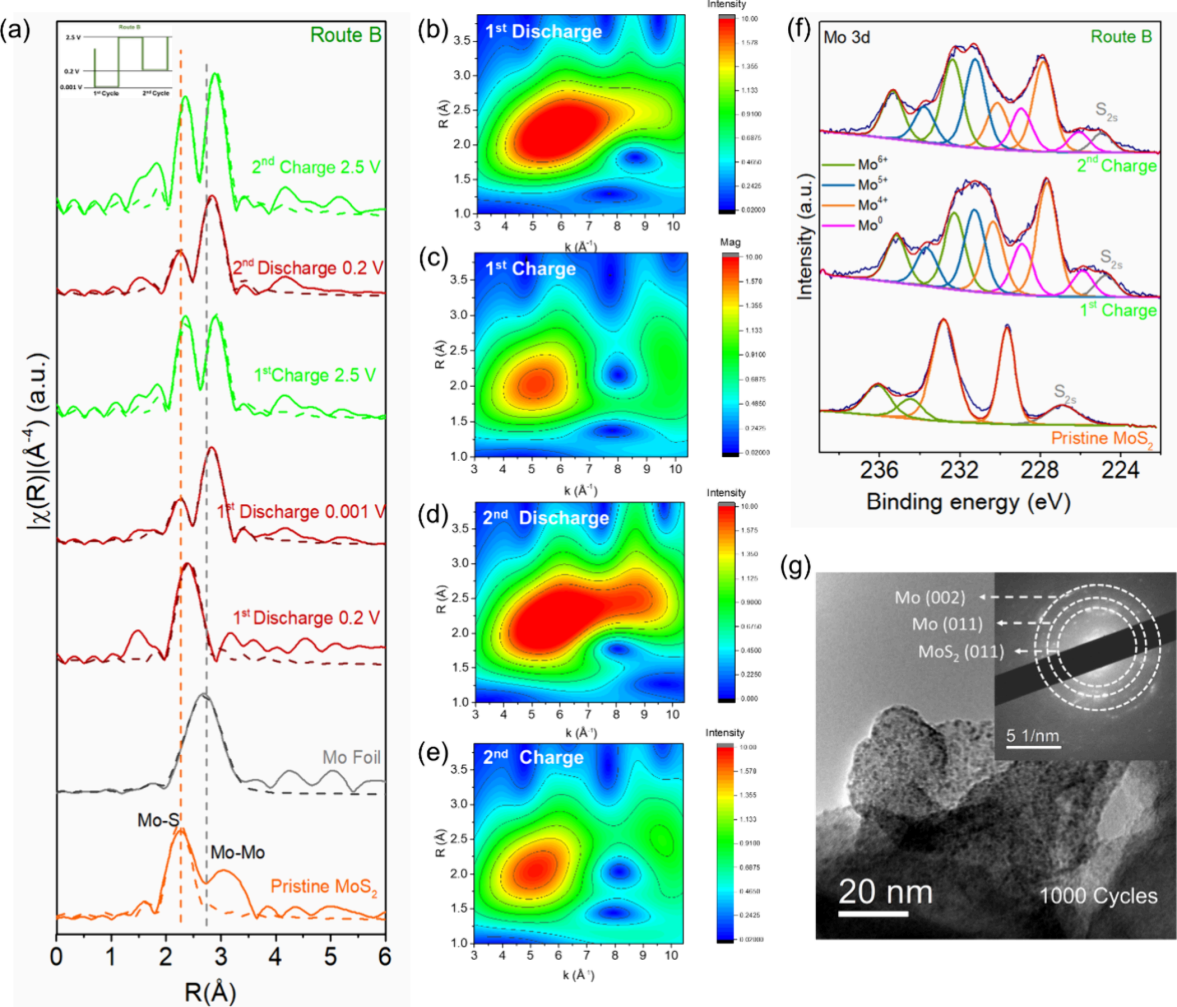
(a) EXAFS spectra of MoS_2_ cycled via Route B during the 1st and 2nd discharge/charge process. Wavelet analysis of the Mo K-edge EXAFS of MoS_2_ cycled via Route B at (b) the 1st discharged state, (c) the 1st charged state, (d) the 2nd discharged state, and (e) the 2nd charged state. (f) Mo 3d XPS spectra of MoS_2_ cycled via Route B in the charged states. (g) TEM image of MoS_2_ cycled via Route B at the charged state (inset: SAED pattern).

The wavelet transform analysis from EXAFS results was performed to further understand this process (Figures 2b–e and S7). The intensity maximum associated with the Mo–S coordination at 5.5 Å^–1^ decreased upon potassiation to 0.2 V (1st discharge 0.2 V, Figure S7c) compared to pristine MoS_2_ (Figure S7a). Further potassiation to 0.001 V (1st discharge of 0.001 V, Figure 2b) caused a significant decrease in the same coordination. But the intensity maximum occurred at 6.1 Å^–1^ with the bond distance of 2.6 Å which corresponds to the Mo–Mo coordination of Mo metal (Figure S7b). Upon depotassiation (1st charge of 2.5 V, Figure 2c), the Mo–S coordination at 5.5 Å^–1^ was less intense compared to its pristine state, which implies partial restoration of MoS_2_. It is worth noting that less intensive metallic Mo–Mo coordination at 6.9 Å^–1^ coexists after depotassiation (1st charge 2.5 V), confirming the presence of Mo metal. In the second cycle, the change of the intensity corresponding to Mo–Mo metal coordination at 6.1 Å^–1^ at potassiation (2nd discharge 0.2 V, Figure 2d) and depotassiation (2nd charge 2.5 V, Figure 2e) can further confirm the existence of the Mo metal.

In addition, the presence of metallic Mo upon depotassiation was proven by Mo 3d XPS spectra. As shown in Figure 2f, the Mo^0^ peaks can be seen at 228.9 (3d_3/2_) and 226.1 eV (3d_5/2_) at the end of the first and second charge, together with Mo^4+^ from the reverse conversion reaction and Mo^5+/6+^ due to partial surface oxidation of the XPS samples. Furthermore, metallic Mo at the depotassiated state of MoS_2_ can be detected even after long-term cycles.^37^ As shown in Figures 2g and S8, dense particles with an average size of 1.5 nm were distributed throughout the MoS_2_ layers. The particles can be characterized as Mo nanocrystals from the selected area electron diffraction (SAED) pattern that shows diffraction spots indexed to the (011) planes of MoS_2_ (ICSD: 98–003–9095) and the (011) and (002) planes of Mo (ICSD: 98–005–1508), confirming the coexistence of MoS_2_ and Mo in the charged state after cycling. In the case of Route A, we also observed the coexistence of metallic Mo^0^ and Mo^4+^ upon depotassiation (Figure S9). Based on these results, it is safe to say that the reverse conversion from Mo^0^ to Mo^4+^ during depotassiation was incomplete, and metallic Mo was present throughout the cycles. However, the consistent presence of metallic Mo would reduce the electrochemically active species in the electrode, and thus, there must be other species that are electrochemically active to contribute to the capacity. This prompted us to investigate the change of S.

Figure 3 shows the change of the S species in MoS_2_ in the first 2 cycles via Route B. The S 2p XPS spectra in the first cycle are shown in Figure 3a. It shows various potassium polysulfides at the discharged state (potassiation), which agrees with previous work.^38,39^ Particularly, the presence of S^2–^, evidenced by the peaks at 160.1 and 158.7 eV, signals the formation of K_2_S and the reduction of Mo^4+^.^38^ What is interesting is that, after depotassiation, peaks of S^0^ appeared at 163.2 and 164.8 eV,^40,41^ and the peak area of S_3_^2–^/S_*x*_^2–^ increased at the expense of the decrease in S_2_^2–^/S^2^. The same changes of the S species were observed in the second cycle (Figure 3b) but with a stronger S^0^ peak intensity. This suggests that during depotassiation, S species was oxidized alongside the partial oxidation of metallic Mo, leading to the formation of S^0^ after charge, which essentially contributes to electron transfer and thus capacity. To ascertain our results, we tested the Raman spectra of the MoS_2_ electrodes at charged states (depotassiation) in the first and second cycles. As shown in Figure 3c, compared with pristine MoS_2_, the signature E^1^_2g_ and A_1g_ vibrational modes largely diminished, signaling the partial restoration of pristine MoS_2_. New peaks evolved at 155 and 244 cm^–1^ in both cycles, corresponding to the torsion and bending vibration modes of sulfur (S_8_)^42^ and therefore further proving the formation of S^0^ after depotassiation. Our analysis of the S species in MoS_2_ points to the fact that, beside Mo, S was electrochemically active after the first potassiation and participated in the oxidation and reduction reactions in the following cycles, effectively making K–S chemistry a part of the K storage mechanism in MoS_2_; in another word, the mechanism is based on both Mo cationic redox and S anionic redox activities. As known, the S conversion process involved in K–S chemistry can lead to the formation of both low-order polysulfides and high-order polysulfides, the latter of which are in the liquid form and can dissolve in electrolytes.^43,44^ If K–S chemistry takes part in the K storage in MoS_2_, we should see high-order polysulfides formed during potassiation dissolving in the electrolyte; as expected, we detected the absorption peaks of S_6_^2–^ and S_5_^2–^ at 300 and 318 nm,^41,43^ respectively, in the UV–vis absorption spectrum of the electrolyte extracted from the separator after the second discharge (Figure 3d). In the case of Route A, since the discharge depth was kept at 0.001 V in all cycles, it is expected that Mo cationic redox and S anionic redox should both contribute to K storage, and the extent to which S anionic redox contributes should be higher than Route B, because deeper discharge depth (0.001 vs 0.2 V) could promote more Mo reduction and as a result, more irreversible Mo oxidation and more S oxidation. As shown in Figure S10, S^0^ was observed after the first and second charge in the XPS (Figure S10a,b) and Raman spectra (Figure S10c), with more noticeable signals after the second charge compared to Route B. Also, S_6_^2–^ and S_5_^2–^ absorption peaks were observed in the UV–vis absorption spectrum (Figure S10d).

**Figure 3:**
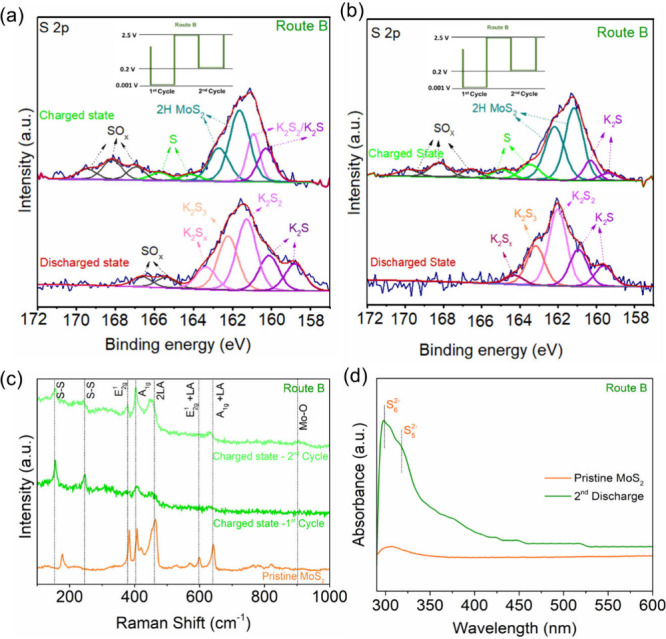
S 2p XPS spectra of MoS_2_ via Route B in the (a) 1st and (b) 2nd cycles. (c) Raman spectra of MoS_2_ cycled via Route B in the charged states. (d) UV–vis absorption spectra of the electrolyte cycled via Route B in the discharged state of MoS_2_.

Our next characterization was to show the cationic and anionic dual redox activity can be sustained over repeated cycles for the K storage in MoS_2_. As shown in Figure S11a, high-order polysulfides S_6_^2–^ and S_5_^2–^ were observed at 245 and 297 nm, respectively, in the UV–vis absorption spectrum after 20 cycles for both Route A and Route B. In the Raman spectra shown in Figure S11b, the presence of the torsion and bending vibration modes of S^0^ proves the reversible transformation of polysulfides to sulfur at the charged state for both routes. Upon combing the HRTEM results shown in Figure 2g, where Mo^0^ was detected at the charged state, the collective results demonstrate the active cationic and anionic dual redox reactions can sustain over repeated cycles, forming dual reaction pathways of the K storage in MoS_2_.

Furthermore, we pointed out in the previous discussion that multiple reactions may occur during K storage in MoS_2_ and controlling the discharge depth could affect the reactions and thus the PIB performance of MoS_2_. Building on our results of cationic and anionic dual redox activity, we carried out postcycling morphological and structural characterizations to understand how controlling discharge depth, the main difference between Route A and Route B, affects the dual reaction pathways and the resulting performance. Constant cycling in the 0.001–2.5 V range (Route A) can maximize capacity because it maximizes the conversion reaction of the Mo species, i.e., cationic redox activity, and potentially maximizes the formation of S^0^, i.e., anionic activity, due to the increasingly irreversible Mo oxidation. This was reflected by the high capacity shown in Figure 1. However, this could result in three consequences: (i) repeatedly destroying the MoS_2_ layered structure at deep discharge depth and reconstructing the structure during charge, (ii) accumulating irreversible discharge products, and (iii) increasing the S^0^ formation and polysulfide dissolution in the electrolyte, all of which are responsible for the capacity decay and deteriorated rate capability via Route A. SEM images (Figures 4a and S12a) show large agglomerations and cracks formed after 1000 cycles, signaling a much thicker (presumably due to continuous formation of solid-electrolyte interphase (SEI)) and stiff surface of the electrode. TEM image (Figure 4b) shows the total collapse of the pristine layered structure (Figure S1e), likely increasing K-ion interparticle diffusion resistance. In contrast, discharging to 0.001 V in cycle1 and raising the discharge depth to 0.2 V from cycle2 (Route B) can balance between maximizing dual cationic and anionic redox activity and minimizing surface deterioration and structural collapse because the dual redox activity is activated in cycle1 and at the same time, the MoS_2_ structure is less prone to collapse from cycle2 after the extent to which the conversion reaction takes place is reduced. The electrode was removed after 1000 cycles. Figures 4c and S12b show a much smoother surface with more defined texture and without cracks via Route B compared to Route A. Layered structure was retained with expanded interlayer spacings (Figure 4d). Additionally, metallic Mo nanoclusters might act as active sites to bind polysulfides and hinder the shuttle effect.^19,45−48^ Collectively, these contributing factors enable long-term cycling stability and rate capability via Route B. Therefore, utilizing cationic and anionic dual redox activity can be advantageous for storing K in MoS_2_, but its execution needs to be balanced.

**Figure 4:**
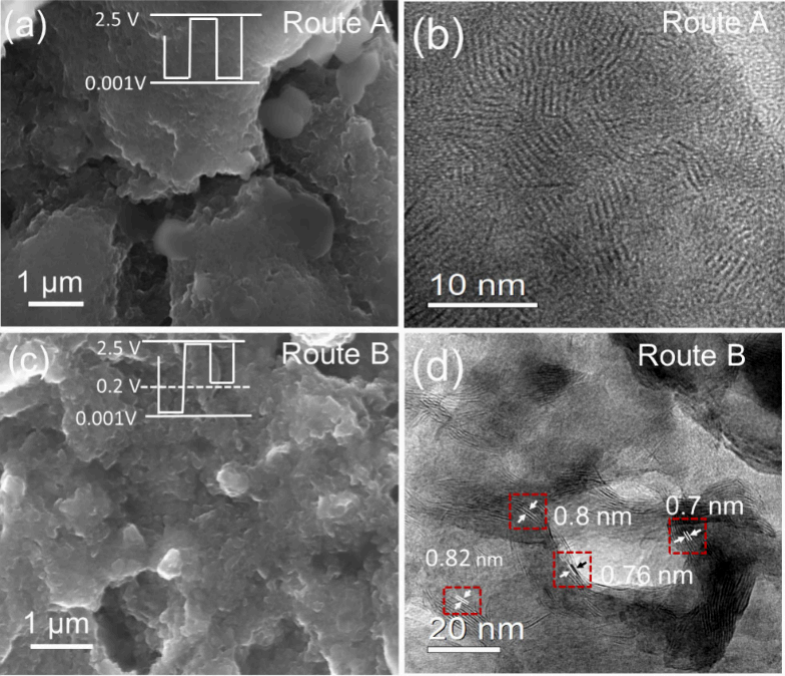
(a, c) SEM and (b, d) TEM images of the MoS_2_ electrodes cycled via (a, b) Route A and (c, d) Route B after 1000 cycles.

With the above results and discussion, we illustrate in Figure 5 the K storage mechanism in MoS_2_. The initial potassiation process at the discharge depth of 0.001 V undergoes a conversion reaction to form a mixture of discharged products, including metallic Mo nanoclusters, potassium (poly)sulfides, and potassium intercalated MoS_2_ (K_*x*_MoS_2_). The subsequent depotassiation process up to 2.5 V does not completely oxidize metallic Mo to higher oxidation states, and a part of metallic Mo remains, which results in a part of potassium sulfides being oxidized to S^0^. In the following cycles, a dual reaction pathway consisting of Mo cationic redox activity and S anionic redox activity takes place, enabling the capacity contribution from transition metal Mo and K–S battery chemistry. Via Route A with which the discharge depth is kept at 0.001 V, the MoS_2_ layered structure is destroyed over cycles and polysulfide dissolution in the electrolyte is increased, leading to fast capacity decay and unstable cyclability. Via Route B with which discharge depth is kept at 0.2 V from cycle 2 onward, severe MoS_2_ structural deterioration is avoided over cycles and polysulfide dissolution in the electrolyte is reduced, enabling stable performance without losing the dual cationic anionic redox reactivity.

**Figure 5:**
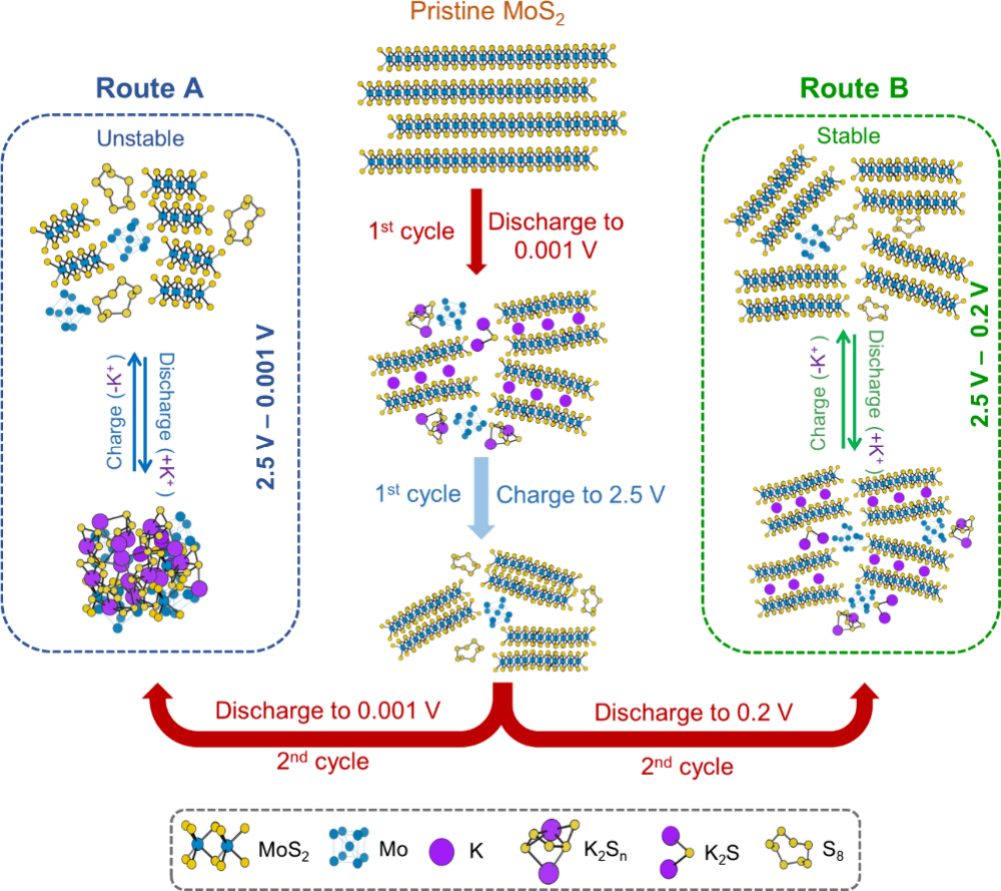
Schematic representation of the mechanism of the K-ion storage process in MoS_2_ via Routes A and B.

In summary, we carried out a mechanistic study on the K storage process in MoS_2_ and revealed that Mo and S conversion reactions occurred simultaneously during (de)potassiation. The process utilized the cationic redox activity of Mo and the anionic redox activity of S simultaneously, which, to the best of our knowledge, was reported for the first time regarding the use of MoS_2_ as an anode material for PIBs. Furthermore, we demonstrated the importance of controlling discharge depth in enabling the advantages of dual redox activity. A balance needs to be taken into consideration between maximizing the dual redox activity and minimizing the structural deterioration of MoS_2_ and the dissolution of polysulfides. To this end, we showed that it is beneficial to deep discharge to 0.001 V in the initial cycle and raise the discharge depth to 0.2 V from the second cycle onward, which enhances capacity retention, cycling stability, and rate capability compared to consistent deep discharge to 0.001 V. We believe our work provides fresh insights into electrochemically storing K-ions in transition metal chalcogenides and an unconventional approach to optimize their PIB performance.

## Supporting Information Available

Materials and methods, characterization of commercial MoS_2_, CV curves and XRD patterns at various stages of discharge/charge via the two routes, fitting of the EXAFS results and wavelet analysis of pristine MoS_2_, Mo foil and MoS_2_ discharged to 0.2 V via Route B, postcycling HRTEM image of MoS_2_ cycled via Route B, XPS/Raman/UV–vis results of MoS_2_ cycled via Route A in the 1st and 2nd cycles, postcycling Raman/UV–vis results and SEM images of MoS_2_ (PDF)
