# Supplementary material for: Cationic and Anionic Dual Redox Activity of MoS2 for Electrochemical Potassium Storage
Source: ACS Mater Lett. 2024 Oct 9;6(11):5031–8. doi: 10.1021/acsmaterialslett.4c01455 (PMC11539095; doi:10.1021/acsmaterialslett.4c01455)
Supplement: Supplementary file 1 — tz4c01455_si_001.pdf [file tz4c01455_si_001.pdf]

Supporting Information

Cationic and anionic dual redox activity of MoS_2 for electrochemical potassium storage

Ajay Piriya Vijaya Kumar Saroja,[1] Yupei Han,[1] Charlie A. F. Nason,[1] Gopinathan Sankar,[1] Pan He,[1] Yi Lu,[1] Henry R Tinker,[1] Andrew Stewart,[1] Veronica Celorrio,[2] Min Zhou,[3] Jiayan Luo,[4] Yang Xu[1,*]

[1]Department of Chemistry, University College London, London WC1H 0AJ, UK

[2]Diamond Light Source, Harwell Science and Innovation Campus, Didcot OX11 0DE, UK

[3] Hefei National Research Center for Physical Sciences at the Microscale, School of Chemistry and Materials Science, University of Science and Technology of China, Hefei, Anhui 230026, China

[4]State Key Laboratory of Metal Matrix Composites, School of Materials Science and Engineering, Shanghai Jiao Tong University, Shanghai 200240, China

Corresponding author: Yang Xu (y.xu.1@ucl.ac.uk)

Materials and Methods

Materials

Commercial MoS_2 was purchased (product number 6694710, BDS chemicals Ltd England) and used as received.

Materials characterization

The crystal structure of MoS_2 was characterized using STOE SEIFERT X-ray diffractometer with Cu Kα source (1.5406 Å, 40 kV, 30 mA). The surface morphology and lattice spacing of MoS_2 were observed by scanning electron microscope (SEM, JEOL JSM 7600) and transmission electron microscope (TEM, JOEL-2100), respectively. Raman spectra were recorded with RENISHAW Raman spectrometer system using 633 nm laser. The chemical states of S and Mo in MoS_2 were evaluated by X-ray photoelectron spectrometer (XPS, Thermo Scientific K-alpha photoelectron spectroscopy) with Al K-α (1486.6 eV) as the X-ray source. The binding energy was calibrated using C_{1s} at 284.8 eV. Data analysis of XPS results was carried out using CasaXPS and high-resolution peaks were deconvoluted using Gaussian-Lorentzian function. UV-vis spectra of the discharged electrodes were measured using UV-Vis spectrophotometer (Shimadzu, UV 2600) in the wavelength range of 200 to 800 nm. The X-ray absorption near edge structure (XANES) spectroscopy measurements of MoS_2 electrodes were carried out at B18 beamline of Diamond Light Source, UK. The Mo K-edge of all the samples were sealed in an aluminum pouch case inside a glove box preventing exposure to air. Mo K-edge spectra were recorded in transmission mode at room temperature. For the post analysis studies, the cycled cells were disassembled in an argon filled glove box and the electrodes were washed with propylene carbonate and dried in the glove box. For the post

analysis of cycled electrode for XPS, KFSI was replaced by KPF_6 as the salt in the electrolyte to avoid the interference of the S species in KFSI for the interpretation of the results.

Electrochemical measurements

MoS_2 electrodes were prepared by mixing 75 wt.% MoS_2, 10 wt.% super P as the conducting agent and 15 wt.% sodium carboxyl methyl cellulose as the binder in DI water to form a slurry. The slurry mixture was coated on a copper foil (9 µm thickness) and dried under vacuum at 60 ˚C overnight. The dried electrodes were punched in the form of discs (diameter 12 mm) and the loading of the active material was 1.5-2 mg cm^{-2}. CR 2032 coin cells were assembled in an argon filled glove box (O_2 < 0.5 ppm, H_2O < 0.5 ppm) using glass fiber B (Whatman Glass Microfiber Filters) as the separator and potassium metal (98%, Thermo Fisher) as the counter and reference electrode. 1 M bis(fluorosulfonyl)imide potassium salt (KFSI) dissolved in 1:1 volume ratio of ethylene carbonate (EC) and diethyl carbonate (DEC) was used as the electrolyte. Galvanostatic charge discharge (GCD) measurements were tested in NEWARE battery cyclers (5V, 20 mA). Cyclic voltammetry (CV) measurements were tested in Biologic VSP potentiostat. Coin cells were rested for 12 h before electrochemical measurements were carried out under different potential ranges including 0.2-2.5 V and 0.001-2.5 V (vs. K^+/K) at various current densities and scan rates.

Characterisation of commercial MoS_2

The XRD pattern of commercial MoS_2 (Figure S1a) shows the characteristic diffraction peaks of 2H MoS_2 (98-003-9095) with high crystallinity. Raman spectrum (Figure S1b) shows peaks appearing at 383 and 407 cm^{-1} corresponding to in-plane Mo-S phonon mode (E^1_{2g}) and out of

plane Mo-S (A_{1g}) vibrational modes, respectively, further demonstrating the 2H phase.[S1] In addition, the peak located at 464 cm^{-1} corresponds to the combination of two Longitudinal Acoustic (LA) modes caused by the resonance of excitation wavelength.[S2] The other peaks represent the combination of LA modes and E^1_{2g}/A_{1g} modes.[S2,S3] To reveal the chemical state of MoS_2, high resolution XPS spectra of Mo 3d and S 2p were deconvoluted. The Mo 3d spectra (Figure S1c) show a main doublet of Mo $3d_{5/2}$ and Mo $3d_{3/2}$ at the binding energies of 232.2 and 229.1 eV, demonstrating the Mo^{4+} state.[S4,S5] The weak doublet at 233.2 and 235.6 eV suggests slight surface oxidation to Mo^{6+}.[S6,S7] The presence of S^{2-} in MoS_2 (Figure S1d) is confirmed by the doublet of S $2p_{3/2}$ and S $2p_{1/2}$ peaks at 162.8 and 163.9 eV, respectively.[S5] SEM images (Figures S1e and S1f) show a layered sheet-like morphology with an average lateral size of ~30 μm and a thickness of ~5 μm. Moreover, the high resolution TEM (HRTEM) image (Figure S1e inset) clearly shows the presence of stacked multilayers with a lattice spacing of 0.62 nm which agrees well with the (002) lattice spacing of 2H MoS_2.[S8] The elemental mapping of MoS_2 shows homogeneous distribution of Mo and S with no impurities and a Mo: S ratio of 1:2.2 (Figure S2).

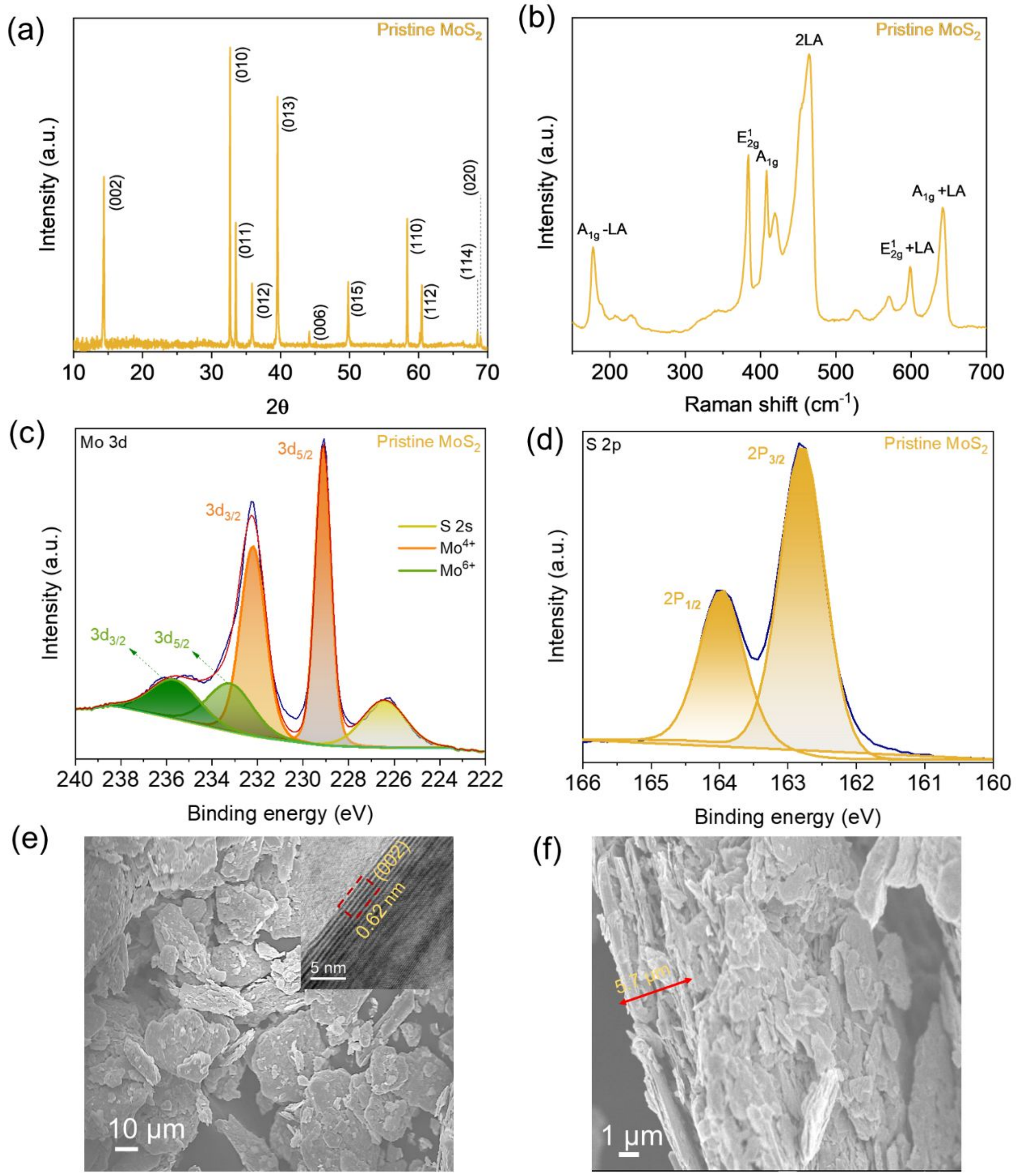

Figure S1. XRD pattern (a), Raman spectrum (b), high-resolution XPS spectra of Mo 3d (c) and S 2p (d), SEM images (e and f), and HRTEM image (inset in e) of commercial MoS_2.

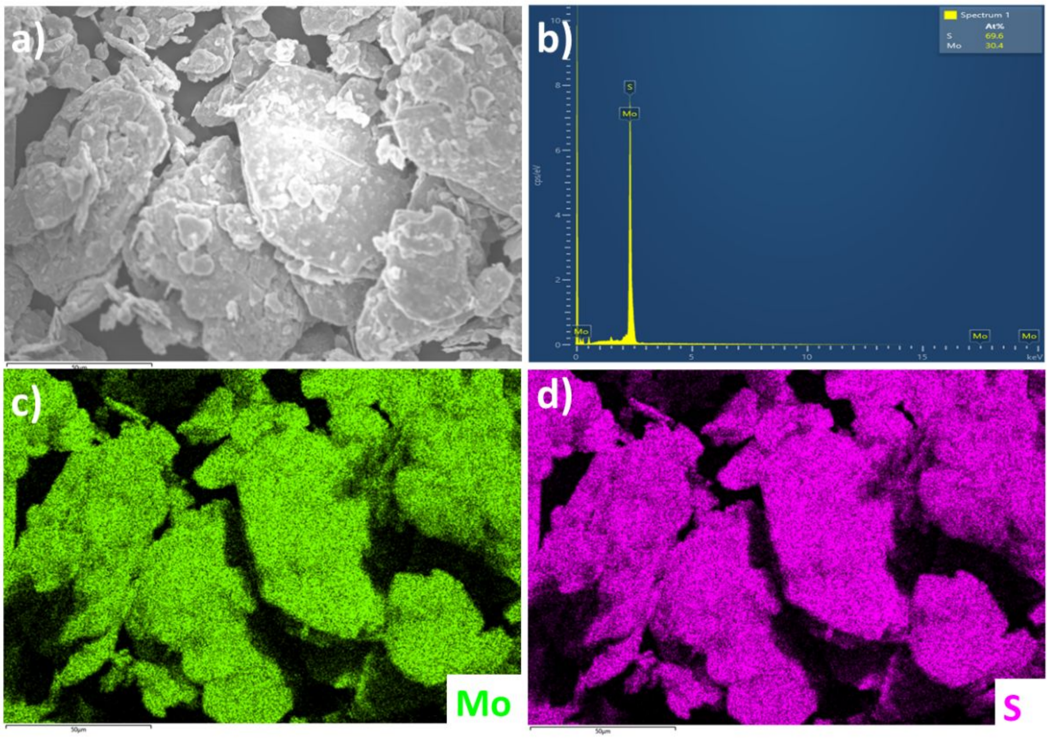

Figure S2. EDS mapping images (a, c nd d) and spectrum (b) of commercial MoS_2.

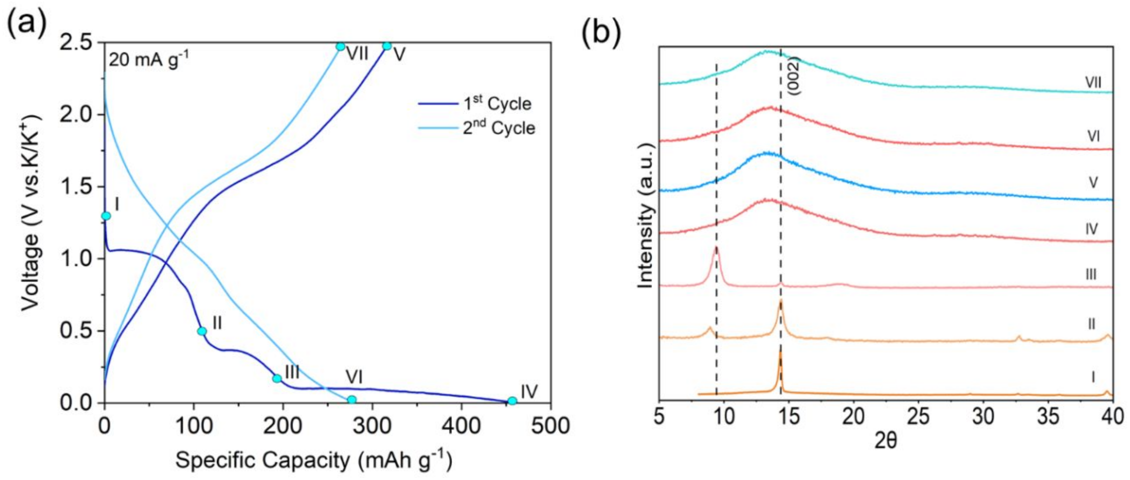

Figure S3. GCD profiles of MoS_2 via Route A in the first 2 cycles (a) and XRD patterns of MoS_2 at various stages of the cycle (b).

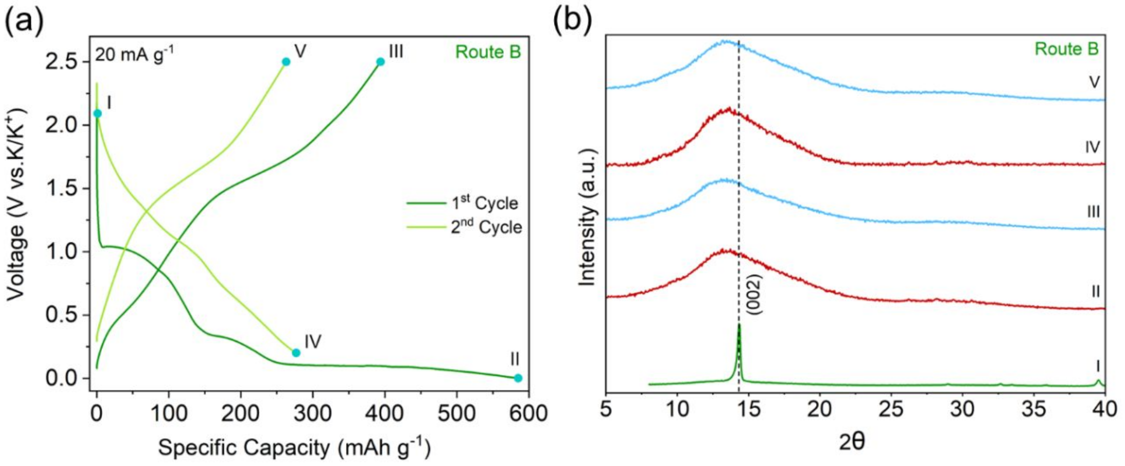

Figure S4. GCD profiles of MoS_2 via Route B in the first 2 cycles (a) and XRD patterns of MoS_2 at various stages of the cycle (b).

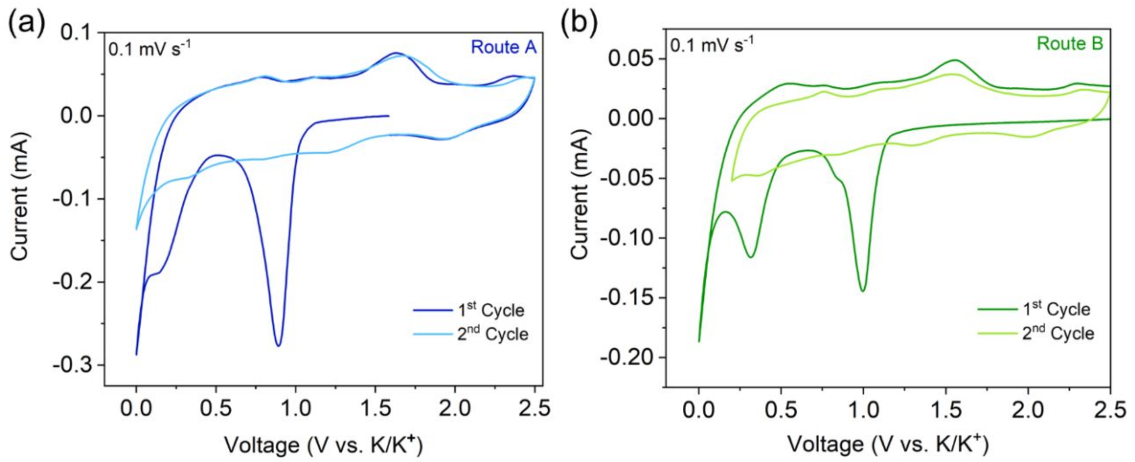

Figure S5. CV curves of MoS_2 via route A (a) and route B (b) at the scan rates of 0.1 mV s^{-1}.

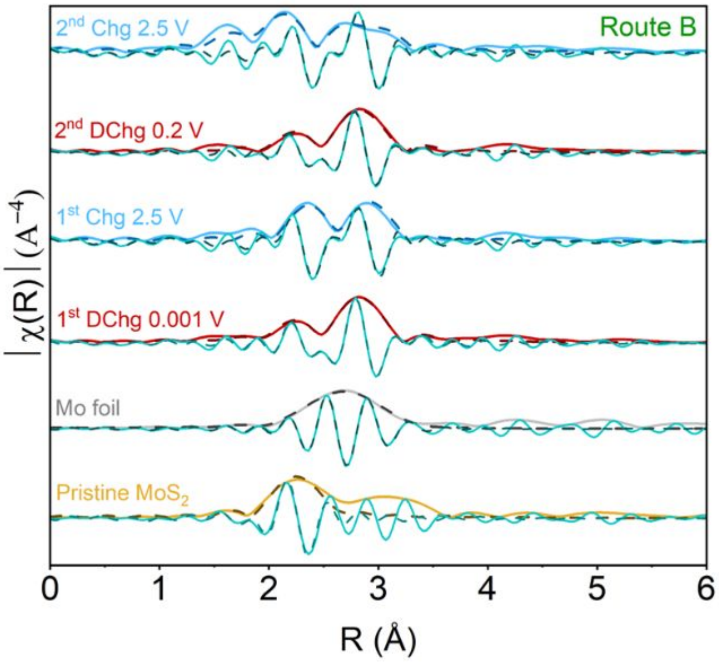

Figure S6. k3-weighted Fourier transform magnitude and imaginary part along with the best-fit model of Mo K-edge EXAFS spectra of MoS_2 at various states of charge and discharge via Route B.

Table S1. Best-fit results for k^3-weighted Mo K-edge EXAFS spectrum data via Route B

Sample	Atom-pair	R (Å)	CN	σ^2	R factor
Pristine MoS_2	Mo-S	2.41	5.9	0.0009	0.0046
	Mo-Mo	3.18	5.9	0.0029	
Mo foil	Mo-Mo at 2.75 (metal)	2.71	8	0.0020	
	Mo-Mo at 3.17 (metal)	3.13	6	0.0012	
1st discharge 0.2 V	Mo-S	2.43	5.7	0.0047	0.0247
	Mo-Mo at 2.75 (metal)	2.77	0	-0.0185	
1st discharge 0.001 V	Mo-S	2.40	2.9	0.0005	0.0230
	Mo-Mo at 2.75 (metal)	2.63	6.0	0.0066	
1st Charge 2.5 V	Mo-S	2.49	5.9	0.0116	0.0461
	Mo-Mo at 2.75 (metal)	2.72	1.9	0.0038	
2nd discharge 0.2 V	Mo-S	2.42	4.2	0.0044	0.0361
	Mo-Mo at 2.75 (metal)	2.65	5.4	0.0059	
2nd Charge 2.5 V	Mo-S	2.50	6.0	0.0114	0.0316
	Mo-Mo at 2.75 (metal)	2.72	1.4	0.0095	

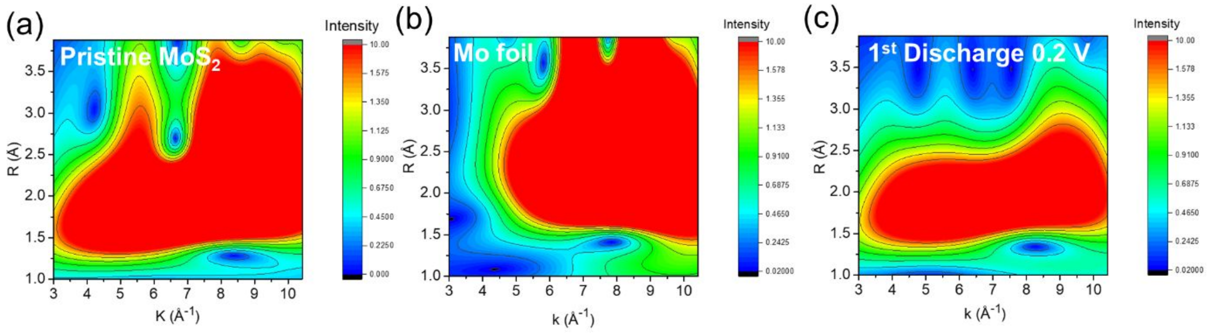

Figure S7. Wavelet analysis of the Mo K-edge EXAFS of MoS_2 cycled via Route B (a) pristine MoS_2, (b) Mo foil, (c) Discharged at 0.2 V (1st cycle).

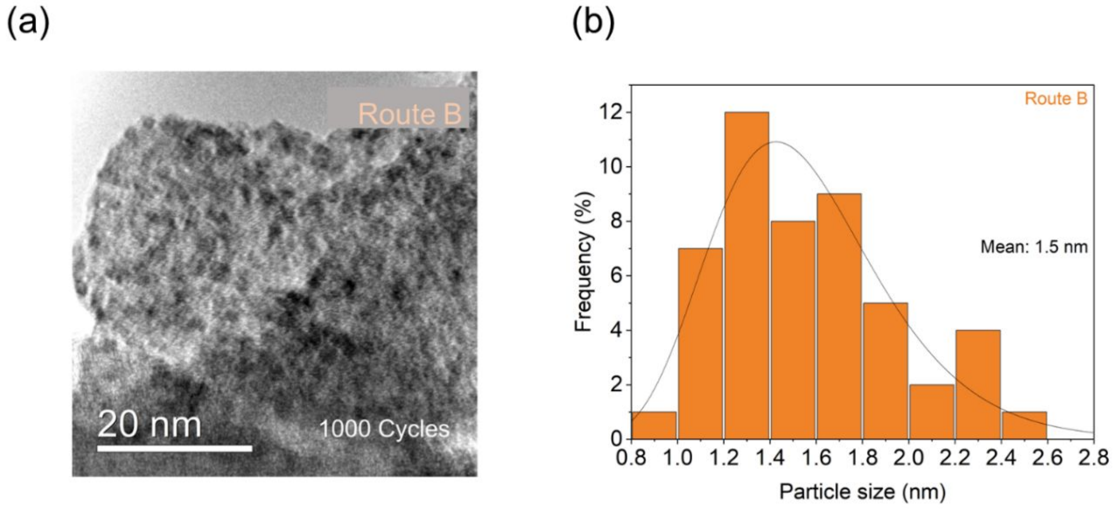

Figure S8. TEM image of MoS_2 cycled via route B showing the distribution of Mo nanoparticles in the charged state (a) and the histogram particle size distribution of Mo (b).

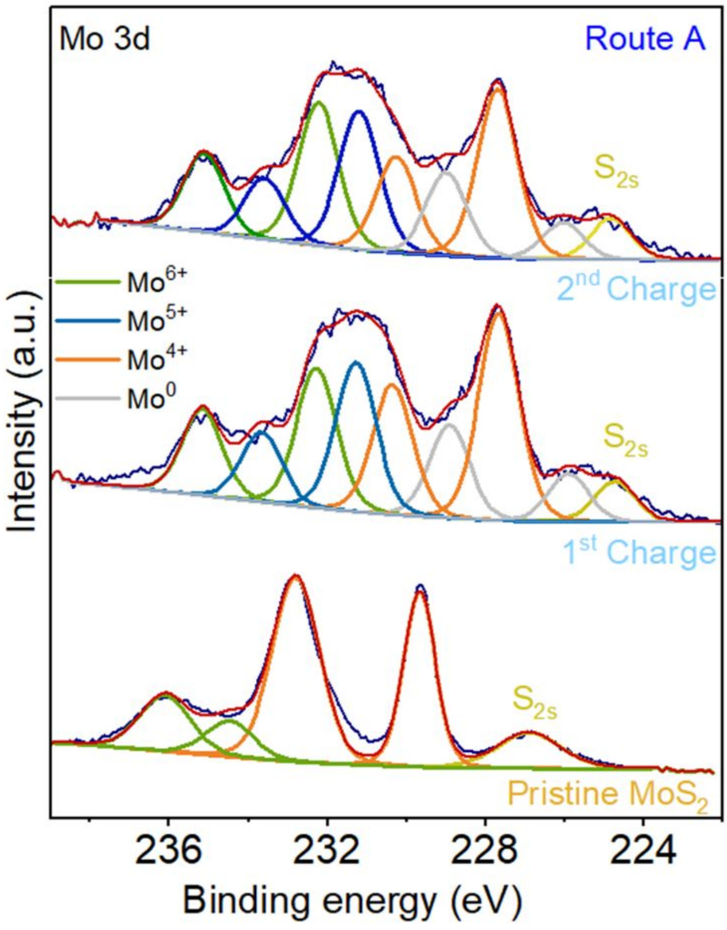

Figure S9. Mo 3d XPS spectra of MoS_2 cycled via route A in the charged states.

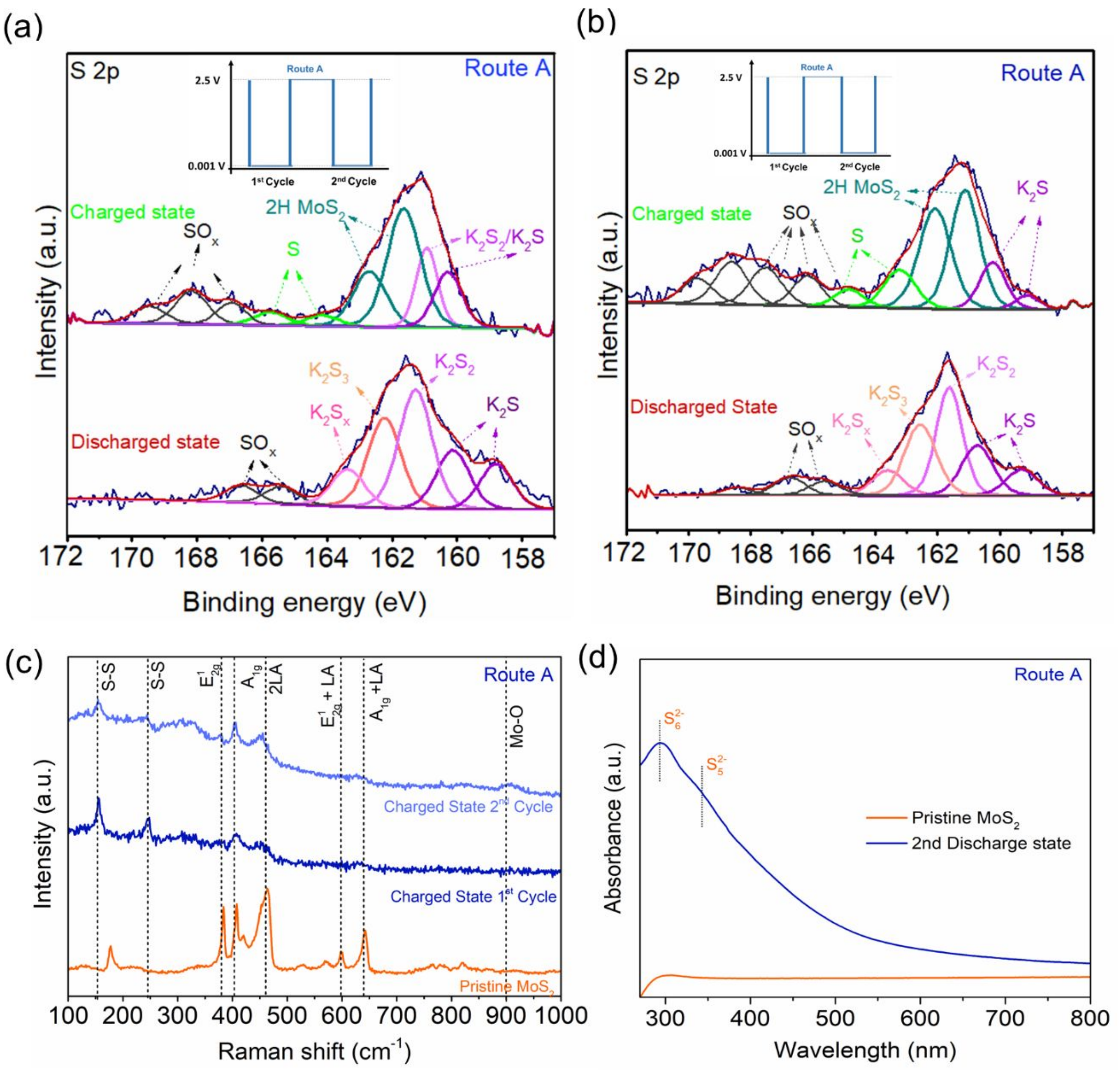

Figure S10. S 2p XPS spectra of MoS_2 via Route A in the 1st (a) and 2nd cycles (b). (c) Raman spectra of MoS_2 cycled via Route A in the charged states. (d) UV-vis absorption spectra of the electrolyte cycled via Route A in the discharged state of MoS_2.

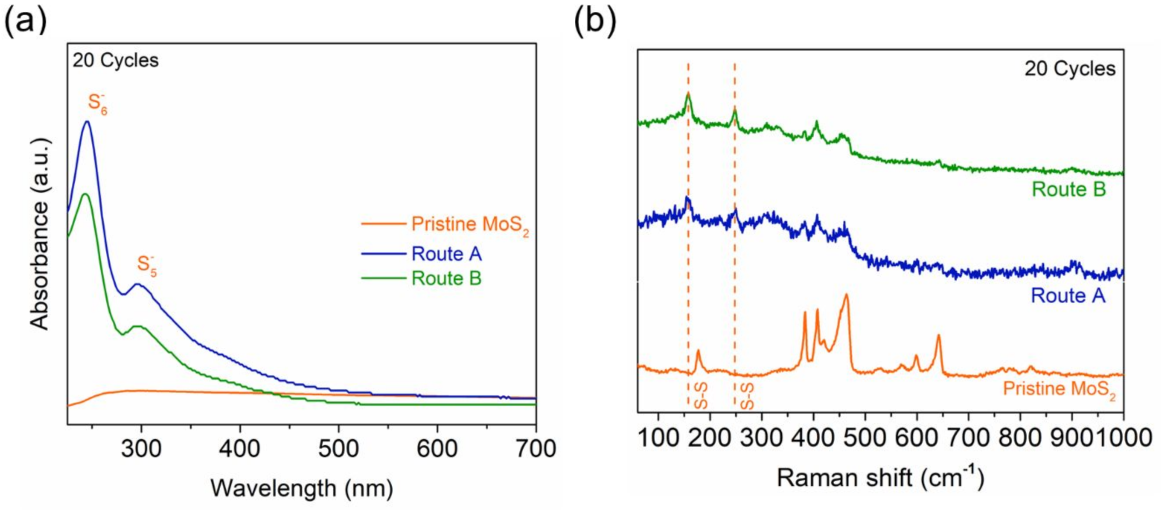

Figure S11. (a) UV-vis absorption spectra of the electrolytes cycled via Routes A and B in the discharged state of MoS_2 after 20 cycles. (b) Raman spectra of MoS_2 cycled via Routes A and B in the charged states after 20 cycles.

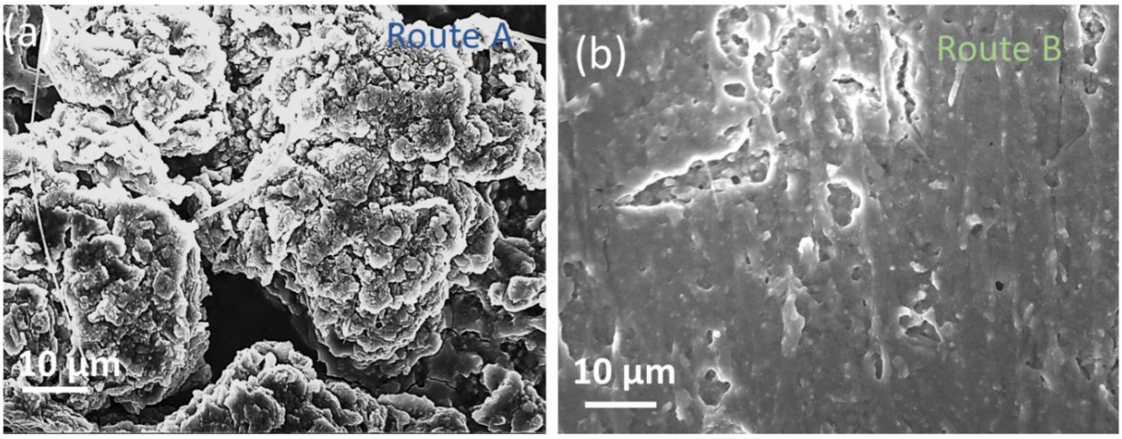

Figure S12. SEM images of the MoS_2 electrodes cycled via Route A (a) and Route B (b) after 1000 cycles.

References

(S1) Xie, Keyu; Yuan, Kai; Li, Xin; Shen,Chao; Liang, Chenglu; Vajtai, Robert; Ajayan, Pulickel; Wei, B. Superior Potassium Ion Storage via Vertical MoS2 Nano-Rose with Expanded Interlayers on Graphene. *Small* **2017**, *13*, 1701471–1701479.

(S2) Fan, J. H.; Gao, P.; Zhang, A. M.; Zhu, B. R.; Zeng, H. L.; Cui, X. D.; He, R.; Zhang, Q. M. Resonance Raman Scattering in Bulk 2H-MX2 (M = Mo, W; X = S, Se) and Monolayer MoS2. *J. Appl. Phys.* **2014**, *115* (5), 053527. https://doi.org/10.1063/1.4862859.

(S3) Placidi, M.; Dimitrievska, M.; Izquierdo-Roca, V.; Fontané, X.; Castellanos-Gomez, A.; Pérez-Tomás, A.; Mestres, N.; Espindola-Rodriguez, M.; López-Marino, S.; Neuschitzer, M.; et al. Multiwavelength Excitation Raman Scattering Analysis of Bulk and Two-Dimensional MoS2: Vibrational Properties of Atomically Thin MoS2 Layers. *2D Mater.* **2015**, *2* (3), 035006–035016. https://doi.org/10.1088/2053-1583/2/3/035006.

(S4) Yao, K.; Xu, Z.; Ma, M.; Li, J.; Lu, F.; Huang, J. Densified Metallic MoS2/Graphene Enabling Fast Potassium-Ion Storage with Superior Gravimetric and Volumetric Capacities. *Adv. Funct. Mater.* **2020**, *30* (24), 2001484–2001492. https://doi.org/10.1002/adfm.202001484.

(S5) Hu, J.; Xie, Y.; Zhou, X.; Zhang, Z. Engineering Hollow Porous Carbon-Sphere-Confined MoS2 with Expanded (002) Planes for Boosting Potassium-Ion Storage. *ACS Appl. Mater. Interfaces* **2020**, *12* (1), 1232–1240. https://doi.org/10.1021/acsami.9b14742.

(S7) Cui, Y.; Zhao, L.; Li, B.; Feng, W.; Cai, T.; Li, X.; Wang, H.; Kong, D.; Fan, Z.; Zhi, L.; et al. Tailored MoS2 Bilayer Grafted onto N/S-Doped Carbon for Ultra-Stable

Potassium-Ion Capacitor. *Chem. Eng. J.* **2022**, *450*, 137815–137825. https://doi.org/10.1016/j.cej.2022.137815.

(S8) Li, J.; Rui, B.; Wei, W.; Nie, P.; Chang, L.; Le, Z.; Liu, M.; Wang, H.; Wang, L.; Zhang, X. Nanosheets Assembled Layered MoS2/MXene as High Performance Anode Materials for Potassium Ion Batteries. *J. Power Sources* **2020**, *449* (November 2019), 227481. https://doi.org/10.1016/j.jpowsour.2019.227481.
